# The role of Frailty Index Laboratory in predicting clinical outcomes in patients with *Clostridioides difficile* infections: a 2020–24 single-centre retrospective study

**DOI:** 10.1093/jacamr/dlaf143

**Published:** 2025-08-08

**Authors:** Giulia Patti, Nicola Veronese, Nicolò De Gennaro, Elda De Vita, Roberta Papagni, Carmen Pellegrino, Angela Amendolara, Vittorio Guerra, Alessandra Vigna, Vito Spada, Mariangela Cormio, Domenica Cassano, Giuliana Metrangolo, Luigi Ronga, Maria Chironna, Stefania Stolfa, Francesco Di Gennaro, Annalisa Saracino

**Affiliations:** Department of Precision and Regenerative Medicine and Ionian Area (DiMePRe-J), Clinic of Infectious Diseases, University of Bari ‘Aldo Moro’, Piazza Giulio Cesare 11, Bari 70124, Italy; Faculty of Medicine, Saint Camillus International University of Health Sciences, Rome, Italy; Department of Precision and Regenerative Medicine and Ionian Area (DiMePRe-J), Clinic of Infectious Diseases, University of Bari ‘Aldo Moro’, Piazza Giulio Cesare 11, Bari 70124, Italy; Department of Precision and Regenerative Medicine and Ionian Area (DiMePRe-J), Clinic of Infectious Diseases, University of Bari ‘Aldo Moro’, Piazza Giulio Cesare 11, Bari 70124, Italy; Department of Precision and Regenerative Medicine and Ionian Area (DiMePRe-J), Clinic of Infectious Diseases, University of Bari ‘Aldo Moro’, Piazza Giulio Cesare 11, Bari 70124, Italy; Department of Precision and Regenerative Medicine and Ionian Area (DiMePRe-J), Clinic of Infectious Diseases, University of Bari ‘Aldo Moro’, Piazza Giulio Cesare 11, Bari 70124, Italy; Department of Precision and Regenerative Medicine and Ionian Area (DiMePRe-J), Clinic of Infectious Diseases, University of Bari ‘Aldo Moro’, Piazza Giulio Cesare 11, Bari 70124, Italy; Department of Precision and Regenerative Medicine and Ionian Area (DiMePRe-J), Clinic of Infectious Diseases, University of Bari ‘Aldo Moro’, Piazza Giulio Cesare 11, Bari 70124, Italy; Department of Precision and Regenerative Medicine and Ionian Area (DiMePRe-J), Clinic of Infectious Diseases, University of Bari ‘Aldo Moro’, Piazza Giulio Cesare 11, Bari 70124, Italy; Department of Precision and Regenerative Medicine and Ionian Area (DiMePRe-J), Clinic of Infectious Diseases, University of Bari ‘Aldo Moro’, Piazza Giulio Cesare 11, Bari 70124, Italy; Department of Precision and Regenerative Medicine and Ionian Area (DiMePRe-J), Clinic of Infectious Diseases, University of Bari ‘Aldo Moro’, Piazza Giulio Cesare 11, Bari 70124, Italy; Department of Precision and Regenerative Medicine and Ionian Area (DiMePRe-J), Clinic of Infectious Diseases, University of Bari ‘Aldo Moro’, Piazza Giulio Cesare 11, Bari 70124, Italy; Department of Precision and Regenerative Medicine and Ionian Area (DiMePRe-J), Clinic of Infectious Diseases, University of Bari ‘Aldo Moro’, Piazza Giulio Cesare 11, Bari 70124, Italy; Department of Interdisciplinary Medicine, Hygiene Section, University of Bari ‘Aldo Moro’, Bari, Italy; Department of Interdisciplinary Medicine, Hygiene Section, University of Bari ‘Aldo Moro’, Bari, Italy; Department of Interdisciplinary Medicine, Hygiene Section, University of Bari ‘Aldo Moro’, Bari, Italy; Department of Precision and Regenerative Medicine and Ionian Area (DiMePRe-J), Clinic of Infectious Diseases, University of Bari ‘Aldo Moro’, Piazza Giulio Cesare 11, Bari 70124, Italy; Department of Precision and Regenerative Medicine and Ionian Area (DiMePRe-J), Clinic of Infectious Diseases, University of Bari ‘Aldo Moro’, Piazza Giulio Cesare 11, Bari 70124, Italy

## Abstract

**Background:**

*Clostridioides difficile* infection (CDI) is considered one of the most significant healthcare-associated infections with significant morbidity and mortality. Frailty, characterized by diminished physiological reserves, has emerged as a critical determinant of poor outcomes. The Frailty Index based on Laboratory tests (FI-Lab), derived from routine laboratory parameters, offers an objective tool for assessing frailty. The primary aim of this study was to assess the efficacy of FI-Lab in predicting mortality and recurrence in CDI hospitalized patients.

**Methods:**

This retrospective study analysed data from 280 patients diagnosed with CDI, hospitalized at the Policlinic of Bari between 2020 and 2024. Frailty was assessed using FI-Lab, based on 35 routine laboratory tests. Primary outcomes included 14- and 28-day mortality, recurrence during hospitalization and recurrence post-discharge. Associations between FI-Lab and outcomes were evaluated.

**Results:**

Of the 280 patients included, 213 survived and 67 died during hospitalization or within 28 days post-infection. Non-survivors had significantly higher FI-Lab scores compared to survivors (0.70 ± 0.15 versus 0.25 ± 0.12, *P* < 0.0001). FI-Lab demonstrated excellent discrimination for mortality at 14 and 28 days, with each 0.10-point increase in FI-Lab associated with elevated mortality risk. Predictive accuracy for recurrence was moderate (AUC = 0.73 for recurrence within 60 days post-discharge). Fidaxomicin use did not significantly reduce mortality or recurrence after adjustment for FI-Lab and comorbidities.

**Conclusions:**

FI-Lab is a predictor of mortality in CDI patients and a valuable tool for early risk stratification. Its utility in predicting recurrences is limited. Prospective studies are warranted to validate these findings and refine therapeutic approaches for high-risk patients.

## Introduction

*Clostridioides difficile* infection (CDI) is one of the most significant healthcare-associated infections (HAIs) in industrialized countries and remains the leading cause of hospital-acquired diarrhoea. CDI is associated with substantial morbidity and mortality, with overall mortality rates estimated to range from 2% to 6%. These rates are significantly higher in elderly patients and in those with comorbidities such as acute renal failure and underlying inflammatory bowel diseases.^1,2^

The epidemiology of *C. difficile* has evolved during the last two decades, mainly due to the emergence of highly virulent and antimicrobial-resistant strains, specifically PCR ribotype 027 and PCR ribotype 078.^3,4^

Fidaxomicin is the recommended first-line therapy for the initial episode of non-severe, severe and severe-complicated/refractory CDI.^5,6^

Fidaxomicin exhibits enhanced preservation of the intestinal microbiota compared with vancomycin while maintaining comparable clinical cure rates and reducing the risk of CDI recurrence.^7–9^

In the absence of fidaxomicin, standard therapy with vancomycin may be administered. Metronidazole is no longer advised as a first-line treatment for CDI due to its poorer clinical cure rate compared to vancomycin^10^ and its high association with recurrence.^11^

Elderly patients are particularly susceptible to severe forms of CDI and unfavourable outcomes due to a combination of immunological decline, nutritional deficits and the burden of comorbidities. Frailty—a clinical condition characterized by diminished physiological reserves and increased vulnerability to stressors—has been identified as a critical factor contributing to adverse health events, including prolonged hospital stays, complications and mortality.^12–14^

Frailty is increasingly recognized as a multidimensional concept encompassing physical, psychological and social dimensions. Importantly, frailty is no longer limited to elderly populations but can affect patients of all ages, depending on their psychophysical and social circumstances.^15^ Various tools and criteria have been developed to assess frailty, with the frailty index (FI) being one of the most widely recognized and utilized. FI quantifies frailty based on accumulated deficits, offering a comprehensive assessment of an individual’s health status. Recently, the use of a Frailty Index based on Laboratory tests (FI-Lab) has been proposed as a novel and objective method to measure frailty.^16,17^

The FI-Lab is constructed using routine laboratory parameters, such as albumin, creatinine, leucocyte count and inflammatory markers, providing a standardized and reproducible approach to assess frailty.^16^ Its integration into clinical practice could enhance risk stratification by offering an objective and accessible measure of frailty.

While several risk factors for poor outcomes in CDI have been identified—including advanced age, disease severity and prior antibiotic use—^18^the inclusion of an objective frailty metric such as the FI-Lab may improve the predictive accuracy for adverse outcomes, thereby optimizing healthcare resources.

The aim of this study was to evaluate the correlation between FI-Lab and clinical outcomes in patients hospitalized with CDI at the University Hospital, Policlinico of Bari.

## Materials and methods

### Study design and participants

This is a retrospective monocentric study conducted at the Policlinico of Bari. From 1 January 1 2020 to 31 October 31 2024, all patients aged 18 years or older and hospitalized and diagnosed with CDI were enrolled in this study. No additional inclusion criteria were applied to ensure the representation of a real-life clinical scenario and capture the full spectrum of CDI cases, ranging from mild to severe presentations.

Patient data were collected retrospectively from medical records. Information included the following:

- Demographics (age, sex and comorbidities)
- Clinical history (prior antibiotic use, recent hospitalizations or immunosuppressive therapy)
- Microbiological findings (toxin detection, strain typing and antibiotic resistance profiles)
- Treatment details (specific CDI therapies and supportive care)

The microbiological diagnosis of CDI was based on the detection of glutamate dehydrogenase (GDH) and toxins A and B in stool samples using lateral flow immunoassays (LFAs) confirmed by PCR assays (Xpert *C. difficile* BT, Cepheid), in accordance with EUCAST and local laboratory protocols. Cases were classified as community-acquired or healthcare-associated, based on established definitions. The study protocol received approval from the Local Ethics Committee in February 2021 (protocol number 02/2021). To ensure compliance with ethical standards, all patient data were fully anonymized and managed in accordance with the Declaration of Helsinki and local data protection regulations.

### Outcomes

The primary outcome was to assess the FI-Lab as an effective clinical instrument for predicting the outcomes of patients with CDI, defined as follows:

- Recurrences during hospitalization and after discharge (60 days)
- Mortality rates at 14 and 28 days
- Occurrence of bacteraemia and candidaemia

Secondary outcome:

- Impact of fidaxomicin on outcomes

### Definitions

**CDI**: presence of diarrhoea (at least three episodes in 24 h not justified by anything else) and a positive test for toxigenic *C. difficile* with toxin expression.

**CDI severe:** one of the following factors at presentation: WBC count of >15 000 cells/mL or a rise in serum creatinine level of >50% above baseline or core body temperature >38.5°C. Additional supporting factors, when available, are distension of the large intestine, pericolonic fat stranding or colonic wall thickening (including low-attenuation mural thickening) at imaging.

**CDI severe-fulminant:** presence of one of the following factors that needs to be attributed to CDI: hypotension, septic shock, elevated serum lactate, ileus, toxic megacolon, bowel perforation or any fulminant course of disease (i.e. rapid deterioration of the patient).^5^

**Charlson Comorbidity Index (CCI):** quantifies overall comorbidity burden.

**Recurrence:** when CDI recurs within 8 weeks after a previous episode, provided the symptoms from the previous episode resolved after completion of initial treatment.^19^

### Exposure: construction of the FI-Lab

The FI-Lab was developed based on 35 laboratory values collected during the first 4 days of hospitalization (Table 1).^16^ These values encompassed blood count, liver function, renal function, pancreatic function, blood glucose, lipid profile, serum electrolytes, coagulation parameters, inflammatory markers and hormonal profiles, including thyroid function and serum vitamin D levels.^16^ Each parameter was assigned a value of 0 for normal results and 1 for abnormal results. The total number of abnormalities was then summed and divided by the total number of tests available for each patient, resulting in a final score ranging from 0 to 1, with higher scores indicating a greater number of abnormalities. No imputation methods were applied, and only two patients with fewer than 10 bio-humoral tests available were excluded. The threshold of 10 missing parameters was established based on prior studies on the FI-Lab, aiming to preserve the accuracy and robustness of the resulting score and to reduce the risk of bias from imputed data.

**Table 1.** Laboratory variables for frailty index^16^

	Item	No frailty (normal)	+1 frailty risk (abnormal)
Routine blood test			
1	Hb (g/L)	115–150 (F)	<110 or >150 (F)
		130–175 (M)	<130 or >175 (M)
2	PLT (*10^9/L)	101–320	<101 or >320
3	WBC (*10^9/L)	3.5–9.5	<3.5 or >9.5
4	NEUT (*10^9/L)	1.8–6.3	<1.8 or >6.3
5	LYMPH (*10^9/L)	1.1–3.2	<1.1 or >3.2
Hepatic function			
6	TBil (umol/L)	5.0–26.0	<5.0 or >26.0
7	DBil (umol/L)	≤8	>8
8	IDBil (umol/L)	≤20	>20
9	ALT (IU/L)	≤40 (F)	>40 (F)
		≤50(M)	>50(M)
10	AST (IU/L)	≤35 (F)	>35 (F)
		≤40(M)	>40(M)
11	ALB (g/L)	32–55	<32 or >55
12	ALP (IU/L)	35–135 (F)	<35 or >135 (F)
		45–125 (M)	<45 or >125 (M)
13	GGT (IU/L)	7–45 (F)	<7 or >45 (F)
		10–60 (M)	<10 or >60 (M)
14	CK (IU/L)	40–200 (F)	<40 or >200 (F)
		50–310 (M)	<50 or >310 (M)
15	LDH (IU/L)	120–250	<120 or >250
Fast blood glucose			
16	GLU (mmol/L)	3.9–6.11	<3.9 or >6.11
Renal function			
17	CREA (umol/L)	41–81 (F)	<41 or >81 (F)
		57–111(M)	<57 or >111 (M)
Blood lipid			
18	TG (mmol/L)	2.3	>2.3
19	CHOL (mmol/L)	≤5.6	>5.6
20	HDL-C (mmol/L)	≥1.15(F)	<1.15(F)
		≥0.9(M)	<0.9(M)
21	LDL-C (mmol/L)	≤4.11	>4.11
Blood electrolyte			
22	Na (mmol/L)	137.0–147.0	<137.0 or >147.0
23	K (mmol/L)	3.5–5.3	<3.5 or >5.3
24	MG (mmol/L)	0.75–1.02	<0.75 or >1.02
25	CA (mmol/L)	2.2–2.7	<2.2 or >2.7
Blood coagulation			
26	INR	0.80–1.30	<0.80 or >1.30
27	Fib (g/L)	2.0–4.0	<2.0 or >4.0
28	D-dimer	<150	>150
Blood respiratory			
29	pO_2_ (mmHg)	>60	<60
30	pH	7.35–7.45	<7.35 or >7.45
31	PCO_2_ (mmHg)	35–45	<35 or >45
32	Lactates (mmol/L)	0.5–2.2	<0.5 or >2.2
33	P/F ratio	>150	<150
Inflammation			
34	CRP (mg/L)	<5	>5
35	Procalcitonin (pg/mL)	<0.05	>0.05
36	Ferritin (ug/L)	24–300	<24 or >330
Hormones			
37	Vitamin D (nmol/L)	50–150	<50 or >150
38	TSH (mIU/L)	0.4–4.0	<0.4 or >4.0
Pancreas			
39	Amilases (UI/L)	<150	>150
40	Lipases (UI/L)	<150	>150

This table lists the laboratory parameters used to construct the FI-Lab. These include easily accessible values such as complete blood count, liver and kidney function markers, inflammatory indices and hormonal and lipid profile. Each parameter is assigned a value of 0 if within the reference range and 1 if outside the reference range.

F, female; M, male; RBC, red blood cell; Hb, haemoglobin; HCT, haematocrit; MCV, mean corpuscular volume; MCH, mean corpuscular haemoglobin; MCHC, mean corpuscular haemoglobin concentration; RDW-CV, red cell distribution width-coefficient of variation; RDW-SD, red cell distribution width-standard deviation; PLT, platelet; WBC, white blood cell; NEUT, neutrophil; LYMPH, lymphocyte; MONO, monocyte; EO, eosinophil; BASO, basophil; TBil, total bilirubin; DBil, direct bilirubin; IDBil, indirect bilirubin; ALT, alanine transaminase; AST, aspartate aminotransferase; TP, total protein; ALB, albumin; ALP, alkaline phosphatase; GGT, gamma-glutamyl transpeptidase; CK, creatine kinase; LDH, lactate dehydrogenase; GLU, glucose; UREA, urea; CREA, creatinine; URIC, uric acid; TG, triglyceride; CHOL, cholesterol; HDL-C, high-density lipoprotein cholesterol-C; LDL-C, low-density lipoprotein cholesterol-C; Na, sodium; K, potassium; Cl, chlorine; MG, magnesium; CA, calcium; P, phosphorus; PT, prothrombin time; INR, international normalized ratio; APTT, activated partial thromboplastin time; Fib, fibrinogen.

### Outcomes: mortality, 14- and 28-day mortality and recurrence

Mortality was recorded through the medical documentation available in the medical records and through death certificates. Overall mortality was posed as primary outcome. We also investigated, as outcomes, mortality at 14 and 28 days after the episode of CDI and the onset of recurrence. Recurrence was defined as the reappearance of clinical symptoms, such as diarrhea (≥3 unformed stools per day for at least 2 consecutive days) and/or abdominal pain, following the resolution of a previous episode.^5^

### Statistical analysis

Patients were divided according to survival status (alive versus deceased) at 28 days, for descriptive purposes. Values for continuous variables were reported as means and SDs or as absolute and relative frequencies (in %) in the two groups. The normality of the continuous variables was assessed using the Kolmogorov–Smirnov test. Continuous variables were compared between the two groups using the Student’s *t*-test for independent samples and categorical variables were compared with the chi-square test, using Fisher’s correction where necessary.

The association between the FI-Lab and mortality (14 or 28 days) was analysed using Cox regression for survival analysis, adjusted for potential confounders. Factors were included in the multivariate analysis as they were statistically different between patients with a *P* value below 0.05. The results were then reported as HR and 95% CI. For outcomes not having a date for event, we used a logistic regression analysis, adjusted for potential confounders and reporting the results as ORs and 95% CI. Since no univocal cut-off was available for the FI-Lab, we used the increase in 0.10 points. We performed an analysis of the accuracy of the FI-Lab in predicting the negative outcomes of our investigation, reporting the data as area AUC and its 95% CI. With this analysis, we chose the best point in terms of sensitivity and specificity using Youden’s Index that was in our cohort equal to 0.50 for all the outcomes, reporting the data as HRs or ORs, according to their nature.

Furthermore, we reported data about the use of fidaxomicin and the outcomes of interest, adjusting the data for age, sex, CCI and FI-Lab.

All analyses were performed using the SPSS 26.0 for Windows (SPSS Inc., Chicago, Illinois). All statistical tests were two-tailed, and statistical significance was assumed for a *P* value of <0.05.

## Results

### Baseline characteristics by survival status (Table 2)

The analysis of baseline characteristics revealed notable differences between survivors and non-survivors among patients affected by CDI. Patients who died during hospitalization (*n* = 67) were significantly older than those who survived (76.8 ± 12.6 years versus 70.6 ± 15.0 years, *P* = 0.002). While gender distribution was comparable between groups, with 47.1% of deceased patients and 43.5% of survivors being male (*P* = 0.60), several comorbidities were more prevalent in the deceased group.

**Table 2.** Baseline characteristics by survival status

Parameter	Alive (*n* = 213)	Dead (*n* = 67)	*P* value
Mean age	70.6 (15.0)	76.8 (12.6)	0.002
Male gender	43.5	47.1	0.60
Italian	43.9	32.4	0.52
*Comorbidities*			
Hospitalization in the previous 3 months	42.2	53.0	0.13
Toxin B	100.0	98.1	0.25
Binary toxin	33.3	26.8	0.29
qezA1	15.9	14.6	0.78
Positive blood culture 15 days before CDI	21.7	12.7	0.07
Positive blood culture 15 days after CDI	30.4	13.6	0.001
No positive blood culture	47.8	73.7	<0.0001
Fungine positive blood culture	10.1	4.2	0.07
COVID-19	21.0	19.6	0.85
Temperature	35.8 (0.7)	36.6 (0.83)	0.22
Ischaemic heart disease	20.2	32.4	0.04
Heart failure	19.2	30.9	0.04
Peripheral artery disease	17.3	17.6	0.95
Cerebrovascular disease	10.3	26.5	0.001
COPD	14.5	26.5	0.02
Diabetes, with complications	5.6	4.5	0.72
IBD	2.9	4.5	0.51
Chronic kidney failure	43.9	79.4	<0.0001
Dialysis	9.4	14.7	0.28
Cholecystectomy	4.4	5.6	0.70
Surgical intervention	18.8	30.0	0.07
Parenteral nutrition	9.8	13.6	0.38
Enteral nutrition	5.8	6.2	0.92
Presence of immunosuppressive conditions	14.7	19.6	0.26
CCI	4.23 (2.29)	5.91 (2.51)	<0.0001
At least one comorbidity	96.2	100	0.11
Median ZAR score, IQR	2 (1–3)	2 (1–2)	0.008
Severe CDI	47.7	66.1	0.04
*Medications*			
Use of antibiotics within 30 days	59.5	68.2	0.21
Use of beta-lactams	13.0	14.6	0.76
Use of cephalosporins	8.7	8.0	0.85
Use of quinolones	5.8	2.8	0.25
Use of clindamycin	0	2.3	0.34
More than one antibiotic	30.4	23.5	0.25
Use of PPI in the last 30 days	18.8	27.2	0.40
Fidaxomicin	20.7	37.7	0.004
Vancomycin	40.6	56.3	0.02
Metronidazole and vancomycin	21.7	22.1	0.96
WBC	11.5 (12.6)	11.5 (8.3)	0.95
CRP	76.1 (69.0)	111.2 (84.2)	0.001
FI-Lab	0.25 (0.12)	0.70 (0.15)	<0.0001

Comparison of demographic and clinical characteristics between CDI survivors and non-survivors. Non-survivors were significantly older and had a higher burden of comorbidities, including chronic kidney disease, cerebrovascular disease and heart failure. Both the CCI and FI-Lab scores were notably higher in non-survivors.

CDI, *Clostridioides difficile* infection; COVID-19, COronaVIrus Disease-2019; COPD, chronic obstructive pulmonary disease; IBD, inflammatory bowel disease; CCI, Charlson Comorbidity Index; PPI, proton pump inhibitor; ZAR score, a prognostic risk index; WBC, white blood cell; CRP, C-reactive protein; FI-Lab, Frailty Index based on Laboratory tests.

Specifically, chronic kidney failure was observed in 79.4% of non-survivors compared to 43.9% of survivors (*P* < 0.0001), and cerebrovascular disease was significantly more frequent in those who died (26.5% versus 10.3%, *P* = 0.001). Similarly, COPD (26.5% versus 14.5%, *P* = 0.02), ischaemic heart disease (32.4% versus 20.2%, *P* = 0.04) and heart failure (30.9% versus 19.2%, *P* = 0.04) were more common among non-survivors.

The CCI was significantly higher in non-survivors, with a mean score of 5.91 ± 2.51 compared to 4.23 ± 2.29 in survivors (*P* < 0.0001). Likewise, the FI-Lab, a tool used to assess frailty, was markedly elevated in the deceased group (0.70 ± 0.15 versus 0.25 ± 0.12, *P* < 0.0001). These findings indicate that older age, greater frailty and a higher burden of comorbidities are associated with mortality in patients with CDI.

The predictive performance of the FI-Lab was assessed for various clinical outcomes, as shown in Table 3. The FI-Lab showed outstanding discriminatory ability for mortality outcomes. For 14-day mortality, the AUC was 0.96 (95% CI: 0.94–0.98, *P* < 0.0001), with a sensitivity of 93% and a specificity of 89%. Similarly, for 28-day mortality, the AUC was 0.99 (95% CI: 0.98–1.00, *P* < 0.0001), with sensitivity and specificity values of 90% and 97%, respectively.

**Table 3.** Accuracy of FI-Lab in predicting negative outcomes in patients affected by *C. difficile*

Outcome	AUC	95% CI	*P* value	Sensitivity	Specificity
Recurrence during hospitalization	0.53	0.40–0.66	0.65	—	—
Recurrence in the 60 days after the discharge	0.73	0.64–0.82	<0.0001	61	83
14-day mortality	0.96	0.94–0.98	<0.0001	93	89
28-day mortality	0.99	0.98–1.00	<0.0001	90	97

Assessment of the predictive power of FI-Lab for CDI patient outcomes using AUC analysis. FI-Lab demonstrated excellent discriminatory ability for mortality, with an AUC of 0.96 for 14-day mortality and 0.99 for 28-day mortality. Its performance in predicting recurrence was moderate post-discharge (AUC = 0.73) but poor during hospitalization (AUC = 0.53).

**Figure 1.**
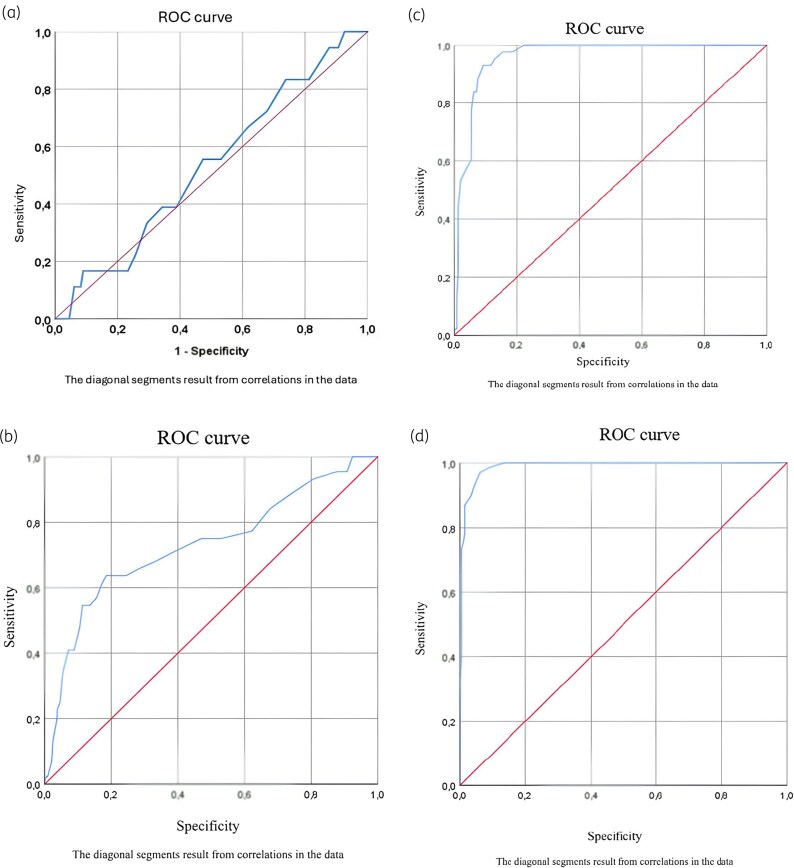
ROC curves of the FI-Lab in predicting recurrence during hospitalization (a), recurrence in the 60 days after the discharge (b), 14-day mortality (c) and 28-day mortality (d). ROC curves illustrating the predictive accuracy of the Frailty Index Laboratory (FI-Lab) for various clinical outcomes. The curve for 28-day mortality is the steepest, approaching the upper left corner, indicating almost perfect discrimination. The curve for recurrence during hospitalization is close to the diagonal, reflecting weak predictive power.

The sensitivity and specificity points were calculated using Youden’s Index, as highlighted in the ROC (Receiver Operating Characteristic) curves shown in Figure 1.

In contrast, the FI-Lab demonstrated moderate accuracy in predicting recurrence within 60 days post-discharge, with an AUC of 0.73 (95% CI: 0.64–0.82, *P* < 0.0001), a sensitivity of 61%, and a specificity of 83%. Notably, its ability to predict recurrence during hospitalization was poor, as reflected by an AUC of 0.53 (95% CI: 0.40–0.66, *P* = 0.65). These results highlight the FI-Lab strength as a mortality predictor while indicating its limitations in forecasting recurrence of CDI.

Data are reported as AUC and the correspondent 95% CIs for FI-Lab as continuous variable. Data about sensitivity and specificity for the different outcomes were reported for a value of FI-Lab = 0.50, the best point according to Youden’s Index.

Figure 1 illustrates the ROC curves for the FI-Lab predictive accuracy for different outcomes. The curves demonstrate excellent discrimination for mortality, particularly for 28-day mortality, where the curve approaches the upper left corner, reflecting near-perfect performance. For recurrence within 60 days post-discharge, the curve is less pronounced, indicating moderate predictive ability. By contrast, the curve for recurrence during hospitalization is near the diagonal, consistent with a lack of predictive power.

#### Association between FI-Lab and adverse outcomes

An increase of 0.10 points in the FI-Lab was associated with a significantly higher risk of adverse outcomes, particularly mortality (Table 4). For 14-day mortality, the HR was 1.90 (95% CI: 1.58–2.28, *P* < 0.0001), while for 28-day mortality, the HR was 1.78 (95% CI: 1.53–2.01, *P* < 0.0001). Additionally, the FI-Lab was strongly associated with recurrence within 60 days post-discharge, with an OR of 1.42 (95% CI: 1.18–1.71, *P* < 0.0001).

**Table 4.** Association between FI-Lab in predicting negative outcomes in patients affected by *C. difficile*

Outcome	Number of events	HR/OR	95% CI	*P* value
Recurrence during hospitalization	18	0.88	0.65–1.20	0.42
Recurrence in the 60 days after the discharge	44	1.42	1.18–1.71	<0.0001
14-day mortality	29	1.90	1.58–2.28	<0.0001
28-day mortality	45	1.78	1.53–2.01	<0.0001
Candidaemia	16	1.12	0.87–1.43	0.39
Bacteraemia	92	1.22	1.06–1.39	0.004

Multivariate analysis results showing the association between FI-Lab and clinical outcomes, including in-hospital and 60-day recurrence, 14- and 28-day mortality and the occurrence of complications such as BSIs and candidaemia. An increase of 0.10 points in FI-Lab was significantly correlated with increased risks of 14- and 28-day mortality. FI-Lab was also associated with BSIs but had limited predictive value for CDI recurrence and candidaemia.

Conversely, the FI-Lab was not significantly associated with recurrence during hospitalization (OR = 0.88, 95% CI: 0.65–1.20, *P* = 0.42) or the occurrence of candidaemia (OR = 1.12, 95% CI: 0.87–1.43, *P* = 0.39). However, it did show a significant association with bacterial bloodstream infections (BSIs), with an OR of 1.22 (95% CI: 1.06–1.39, *P* = 0.004). These findings emphasize the FI-Lab as a robust predictor of mortality and late recurrence, suggesting its utility in identifying high-risk patients.

Data are reported as HRs with their 95% CIs for mortality outcomes and as ORs for the other outcomes, for an increase of 0.10 points in the FI-Lab. The results are adjusted for age, sex and comorbidities (represented as CCI).

**Figure 2.**
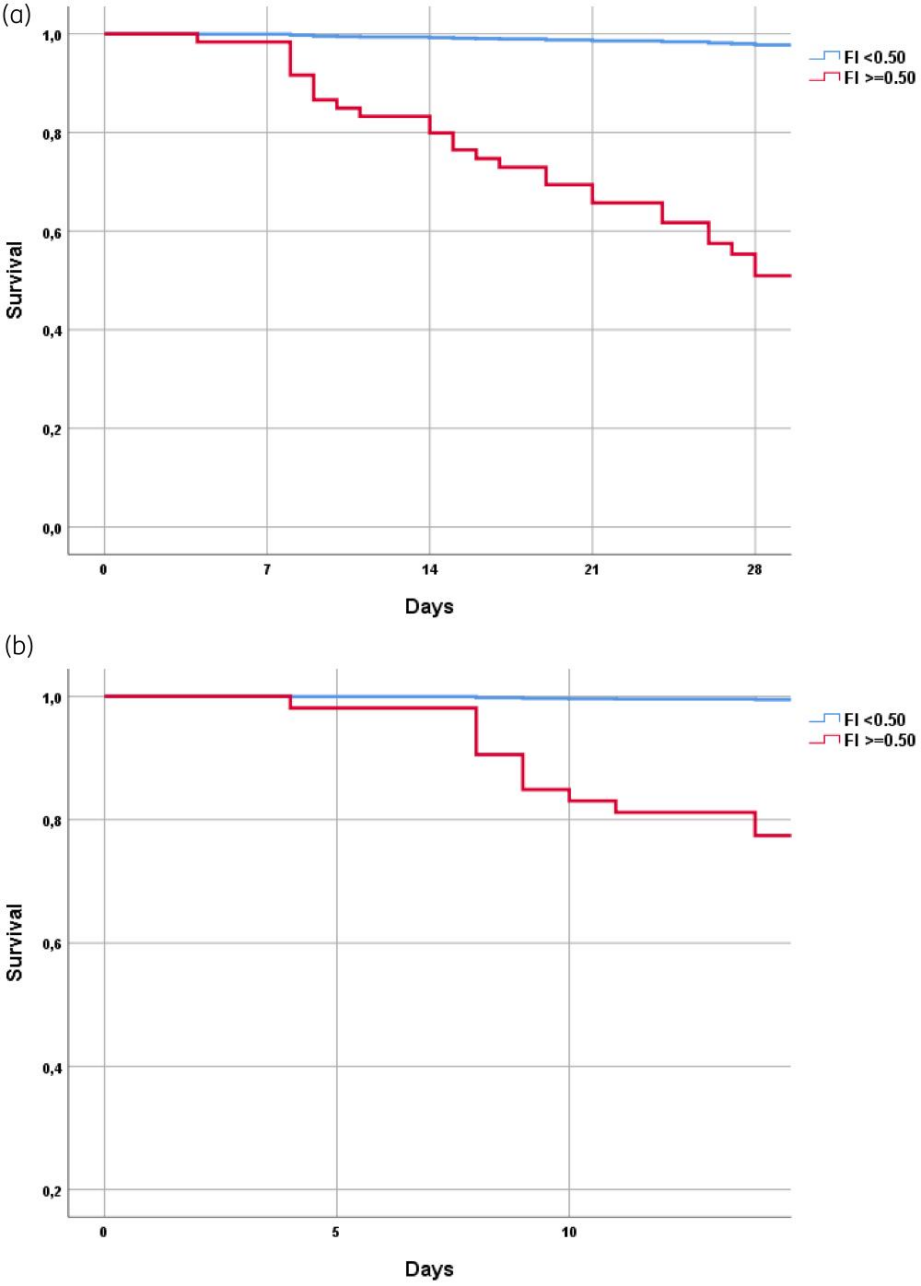
Survival curves in frailer people (FI-Lab > 0.50) for 28-day mortality (a) and 14-day mortality. Two hundred fourteen patients had a FI-Lab ≤ 0.5, and 68 patients had a FI-Lab > 0.5. Kaplan–Meier survival curves illustrating the relationship between frailty, as measured by FI-Lab, and mortality. Patients with FI-Lab scores above 0.50 exhibit significantly lower survival probabilities. The most significant decline is observed within the first 14 days, highlighting the role of frailty in predicting adverse outcomes in patients with CDI.

The Kaplan–Meier survival curves in Figure 2 show a clear association between frailty (FI-Lab > 0.50) and mortality. Patients with higher frailty scores experienced significantly poorer survival, with sharp declines in survival probability within the first 2 weeks for 14-day mortality and over the first month for 28-day mortality. These findings further support the role of frailty as a key determinant of outcomes in CDI patients.

#### Impact of fidaxomicin on outcomes

The use of fidaxomicin was evaluated as a potential factor influencing clinical outcomes (Table 5). However, no significant associations were observed between fidaxomicin treatment and reduced risks of mortality or recurrence. For 14-day mortality, the HR was 1.00 (95% CI: 0.45–2.23, *P* = 0.99), while for 28-day mortality, the HR was 0.70 (95% CI: 0.35–1.42, *P* = 0.32). Similarly, fidaxomicin did not significantly reduce the risk of recurrence during hospitalization (OR = 1.33, 95% CI: 0.39–4.55, *P* = 0.64) or within 60 days after discharge (OR = 0.96, 95% CI: 0.34–2.67, *P* = 0.93).

**Table 5.** Association between fidaxomicin in predicting negative outcomes in patients affected by *C. difficile*

Outcome	HR/OR	95% CI	*P* value
Recurrence during hospitalization	1.33	0.39–4.55	0.64
Recurrence in the 60 days after the discharge	0.96	0.34–2.67	0.93
14-day mortality	1.00	0.45–2.23	0.99
28-day mortality	0.70	0.35–1.42	0.32
Candidaemia	0.27	0.03–2.19	0.22
Bacteraemia	1.24	0.62–2.49	0.54

Analysis of the impact of fidaxomicin on clinical outcomes, including mortality and recurrence. Fidaxomicin did not show a statistically significant association with improved survival or reduced recurrence when adjusted for age, sex, comorbidities and frailty.

These findings suggest that, when adjusted for age, sex, comorbidities and frailty, fidaxomicin does not independently influence mortality or relapse outcomes in patients with CDI.

Data are reported as HRs or ORs with their 95% CIs for fidaxomicin versus other treatments specific for CD [reference, HR = 1]. The results are adjusted for age, sex, comorbidities (represented as CCI) and an increase of 0.10 points in the FI-Lab.

## Discussion

Our study, which included 280 patients, underscores the importance of factors such as advanced age, frailty and comorbidities in predicting adverse outcomes in patients with CDI. Among the non-survivors (*n* = 67), a significantly higher mean age and burden of comorbidities were observed compared to survivors. Comorbidities most strongly associated with mortality included chronic renal failure (79.4%), cerebrovascular disease (26.5%), COPD (26.5%), ischaemic heart disease (32.4%) and heart failure (30.9%). Both the CCI and the FI-Lab were significantly elevated in the deceased group.

Higher FI-Lab scores demonstrated a strong association with increased mortality, confirming its role as a sensitive and accurate tool for predicting short-term mortality at both 14 days (AUC = 0.96) and 28 days (AUC = 0.99). These findings highlight the potential of the FI-Lab, derived exclusively from routine bio-humoral tests, to facilitate early identification of frail patients and enable timely, targeted interventions. Unlike clinical frailty indices, the FI-Lab reliance on easily accessible laboratory data makes it a practical and broadly applicable tool in various healthcare settings.

However, the predictive performance of the FI-Lab was less robust for CDI recurrence. While the AUC for predicting recurrences during hospitalization was 0.53, its accuracy improved when predicting recurrences within 60 days post-discharge (AUC = 0.73). This suggests that factors beyond baseline frailty, such as therapeutic management or hospital environment, may play a more prominent role in recurrence during hospitalization. Moreover, we can hypothesize that FI-Lab is a stronger determinant of mortality than other negative outcomes among older people.

Although the FI-Lab did not predict early CDI recurrence or the occurrence of candidaemia, it was significantly associated with BSIs, further reinforcing its utility in identifying patients at higher risk of severe outcomes.

Our findings provide evidence of a significant association between CDI and the development of secondary infections, particularly nosocomial BSIs. A particularly relevant aspect is the high mortality observed in patients affected by this complication. The association between BSI and mortality remained significant even after adjustment for frailty, as measured by the FI-Lab, suggesting that BSIs may act as independent amplifiers of frailty-related risk.

From a clinical perspective, these data underscore the importance of close surveillance during the first 2–4 weeks following CDI diagnosis, a period during which the risk of BSI appears to be especially high. In this context, early blood culture collection and active monitoring for invasive complications may contribute to improved outcomes, particularly in frail patients or those with severe or recurrent CDI.

The mechanisms underlying this association are likely multifactorial and include disruption of intestinal mucosal integrity—particularly that caused by hypervirulent *C. difficile* strains (notably ribotype 027)—as well as intestinal dysbiosis secondary to prolonged antibiotic therapy. Moreover, the high incidence of multidrug-resistant organisms (such as ESBL- and carbapenemase-producing Enterobacteriaceae) among BSI isolates reinforces the role of the gastrointestinal tract as a reservoir of opportunistic pathogens in frail patients with frequent healthcare exposures.^20^

In light of these findings, we believe that integrating objective frailty assessment tools, such as the FI-Lab, into routine clinical practice could provide valuable support for risk stratification and optimization of therapeutic decision-making. Specifically, the FI-Lab may help identify patients with CDI who are at increased risk of invasive complications, thereby guiding more personalized choices regarding monitoring, diagnostic workup and targeted antimicrobial therapy—particularly in resource-limited settings or where access to high-cost treatments is restricted.

Our findings are consistent with previous studies in other clinical contexts. For example, studies on elderly patients hospitalized with COVID-19 and critically ill patients with heart failure have demonstrated the strong predictive value of the FI-Lab for short- and long-term mortality.^21,22^ These results confirm that frailty, as assessed through biomarkers, is a powerful indicator of poor prognosis across diverse conditions.

Although frailty is traditionally associated with advanced age, emerging evidence suggests that it can also affect younger patients, particularly those with chronic diseases, immunosuppression or malnutrition. Several studies have shown that biological age, more than chronological age, can be a more accurate predictor of unfavourable outcomes in various clinical contexts, including infections. In this sense, the FI-Lab, based on objective laboratory parameters, represents a practical and scalable tool for assessing frailty even in younger populations that may not be clinically recognized as frail. The early identification of these patients can facilitate timely interventions, targeted monitoring and optimized therapeutic strategies. Extending the use of FI-Lab beyond geriatric cohorts could therefore improve risk stratification across a broader age spectrum and contribute to a more personalized approach to care.

The role of fidaxomicin in CDI management was also examined. Adjusted analyses revealed no significant association between fidaxomicin use and reduced risks of mortality or recurrence. Although fidaxomicin is favoured over vancomycin in the 2021 ESCMID and IDSA guidelines due to its potential to prevent recurrences,^5,6^ our data suggest that its benefits may be influenced by factors such as patient frailty, timing of therapy initiation and treatment duration. The lack of a direct mortality benefit aligns with the limited focus on mortality in existing guidelines, despite the established link between CDI recurrence and increased mortality.^23^

Our results suggest that the FI-Lab could explain the apparent lack of an independent benefit of fidaxomicin after adjusting for frailty and other confounders. In frail and vulnerable patients, a holistic approach addressing frailty and comorbidities, alongside antibiotic treatment, is critical for improving outcomes.

The FI-Lab serves as an objective metric derived from standard laboratory values, effectively identifying the most vulnerable individuals who may warrant a more aggressive or timely therapeutic intervention. The strategic application of fidaxomicin, informed by risk assessment using FI-Lab, may enhance therapeutic effectiveness and optimize resource distribution, particularly in patients with a heightened risk of problems. Ultimately, we assert that the FI-Lab could facilitate a more judicious selection of patients for expensive therapies, hence enhancing individualized and sustainable healthcare.

The findings of our study should be interpreted considering its limitations. First, as a retrospective and monocentric analysis, it may be subject to selection and confounding biases. Second, the study population included patients with severely compromised clinical states, potentially limiting the generalizability of our results. Third, the FI-Lab was only evaluated at baseline, without accounting for dynamic changes in bio-humoral parameters, such as blood count or renal function, during hospitalization. Finally, our findings may have been influenced by the circulation of specific endemic *C. difficile* strains within the study setting, which could further limit their applicability to other geographical or epidemiological contexts.

In conclusion, the FI-Lab is a valuable tool for predicting mortality in patients with CDI. Its ability to identify the frailer subjects renders it a reliable screening instrument to guide targeted therapy strategies and facilitate a more expedited therapeutic approach and timely intervention in the event of inadequate improvement in the clinical condition due to the risk of complications.

Additionally, its effectiveness is derived from the fact that it is exclusively composed of routine laboratory tests, which do not result in additional costs. Consequently, it is particularly advantageous in a variety of hospital environments, including those with limited resource. This attribute renders it advantageous for daily clinical practice, as it facilitates seamless integration into standard hospital workflows. It is a practical and economically sustainable approach to risk stratification in hospitalized patients with CDI that is particularly useful in contexts with limited resources or a lack of more complex tools for frailty assessment, due to its simplicity and objectivity, which facilitate broader implementation and scalability.

The FI-Lab score may be a valuable adjunctive tool for risk stratification in patients with CDI, but its predictive value must be interpreted in conjunction with other clinical parameters and cannot be considered in isolation.

However, its utility in predicting recurrences is limited, underscoring the need for complementary approaches to recurrence prevention.

The lack of a significant benefit associated with fidaxomicin in reducing mortality or recurrence highlights the necessity of a holistic therapeutic approach, combining antibiotic treatment with interventions targeting frailty and comorbidities.

Beyond its predictive significance, the FI-Lab may serve as a valuable instrument to aid the clinical decision-making process at multiple phases of managing patients with CDI. An elevated score may warrant an early referral to specialists to enhance the therapeutic strategy for the most susceptible patients. Furthermore, the FI-Lab can facilitate an enhancement of care, such as through increased monitoring, access to greater intensity care environments or the prompt implementation of supportive therapies. Ultimately, the FI-Lab might be included into antimicrobial stewardship programmes, aiding in the identification of patients at elevated risk for infection complications and facilitating more precise therapeutic decisions regarding the type, duration and intensity of antibiotic treatment. The incorporation of the FI-Lab into routine clinical practice could thus aid in forecasting outcomes while also customizing and enhancing care.

While the FI-Lab may be predictive of mortality more broadly in hospitalized patients, our findings are specific to patients with CDI, and conclusions beyond this population require cautious interpretation and further studies.

Prospective, multicentric studies with comprehensive frailty stratification are warranted to confirm these findings and explore the broader applicability of the FI-Lab in other clinical settings. Additionally, examining subgroups of patients with moderate to severe frailty may reveal populations that could derive enhanced benefits from fidaxomicin or alternative therapeutic strategies. Certainly, prospective validation, integration with clinical frailty indices and the consideration of FI-Lab calculation not only at hospital admission but also during hospitalization could contribute to a more accurate assessment of patient status and help guide therapeutic decision-making through more personalized treatment strategies. In addition, future cohort studies with a control group for CDI are necessary, to better understand the role of FI in determining mortality risk.
